# Structures and Biogenesis of Fallaxosides D_4_, D_5_, D_6_ and D_7_, Trisulfated Non-Holostane Triterpene Glycosides from the Sea Cucumber *Cucumaria fallax*

**DOI:** 10.3390/molecules21070939

**Published:** 2016-07-20

**Authors:** Alexandra S. Silchenko, Anatoly I. Kalinovsky, Sergey A. Avilov, Pelageya V. Andryjaschenko, Pavel S. Dmitrenok, Vladimir I. Kalinin, Ekaterina A. Chingizova, Kirill V. Minin, Valentin A. Stonik

**Affiliations:** 1 G.B. Elyakov Pacific Institute of Bioorganic Chemistry, Far Eastern Branch of the Russian Academy of Sciences, Pr. 100-letya Vladivostoka 159, 690022 Vladivostok, Russia; sialexandra@mail.ru (A.S.S.); kaaniv@piboc.dvo.ru (A.I.K.); Avilov-1957@mail.ru (S.A.A.); pandriyashchenko@inbox.ru (P.V.A.); paveldmt@piboc.dvo.ru (P.S.D.); kalininv@piboc.dvo.ru (V.I.K.); martyyas@mail.ru (E.A.C.); 2 P.P. Shirshov Institute of Oceanology of the Russian Academy of Sciences, Nakhimovsky Pr., 36, 117997 Moscow, Russia; kirill.minin569@gmail.com

**Keywords:** sea cucumbers, *Cucumaria fallax*, triterpene glycosides, fallaxosides, non-holostane glycosides

## Abstract

Four new trisulfated triterpene glycosides, fallaxosides D_4_ (**1**), D_5_ (**2**), D_6_ (**3**) and D_7_ (**4**) have been isolated from the sea cucumber *Cucumaria fallax* (Cucumariidae, Dendrochirotida). The structures of the glycosides have been elucidated by 2D NMR spectroscopy and HRESIMS. All the glycosides have the lanostane aglycones of a rare non-holostane type with 7(8)-, 8(9)- or 9(11)-double bonds, one or two hydroxyl groups occupying unusual positions in the polycyclic nucleus and shortened or normal side chains. The pentasaccharide carbohydrate moieties of **1**–**4** have three sulfate groups. The cytotoxic activity of glycosides **1**–**4** against the ascite form of mouse Ehrlich carcinoma cells and mouse spleen lymphocytes and hemolytic activity against mouse erythrocytes have been studied.

## 1. Introduction

Triterpene glycosides are characteristic metabolites of sea cucumbers (class Holothurioidea, phylum Echinodermata). Majority of them contain holostane type aglycones (lanostane derivatives with 18(20)-lactone) [1], but rare non-holostane aglycones, i.e., lanostane derivatives have not 18(20)-lactone [2,3,4,5,6,7].

Recently we have started studies on the Far-Eastern sea cucumber *Cucumaria fallax* [6] which contains exclusively non-holostane oligoglycosides having unusual double bond positions and uncommon sites of oxidation in their aglycone moieties. Herein we report the isolation of four new trisulfated glycosides, fallaxosides D_4_–D_7_ (compounds **1**–**4**) with earlier unknown aglycones and their structures, established by analysis of ^1^H-, ^13^C-NMR and 2D NMR (^1^H−^1^H COSY, 1D TOCSY, HMBC, HSQC, ROESY) spectra and confirmed by HRESI mass spectrometry. The biogenesis of these unusual metabolites is also discussed.

## 2. Results and Discussion

The sea cucumbers were extracted with 70% ethanol under reflux during 5 h. The concentrated extract was sequentially submitted to the column chromatography on Polychrom-1 (powdered Teflon) in H_2_O→50% ethanol in order to eliminate salts and polar impurities and on Si gel using CHCl_3_/EtOH/H_2_O (100:125:25 and 100:150:50) as mobile phases to obtain the fraction containing polar trisulfated pentaosides (glycosides belonging to the group A_7_). Further separation of the fraction by HPLC on a semi-preparative reversed phase column using MeOH/H_2_O/NH_4_OAc (1 M water solution) as mobile phase in ratio 60/39/1 gave the subfractions A_7_I–A_7_V. Each of the subfractions was rechromatographed using HPLC. The HPLC of subfraction A_7_I with the same solvent system in ratio of 35/64/1 gave fallaxoside D_4_ (**1**) and fallaxoside D_5_ (**2**). The HPLC of subfraction A_7_II using the solvent system in ratio of 50/49/1 followed by 45/54/1 and 47/51/2 gave fallaxoside D_7_ (**4**). The HPLC of subfraction A_7_V with the same solvents in ratio of 58/41/1 gave fallaxoside D_6_ (**3**).

**Scheme 1:**
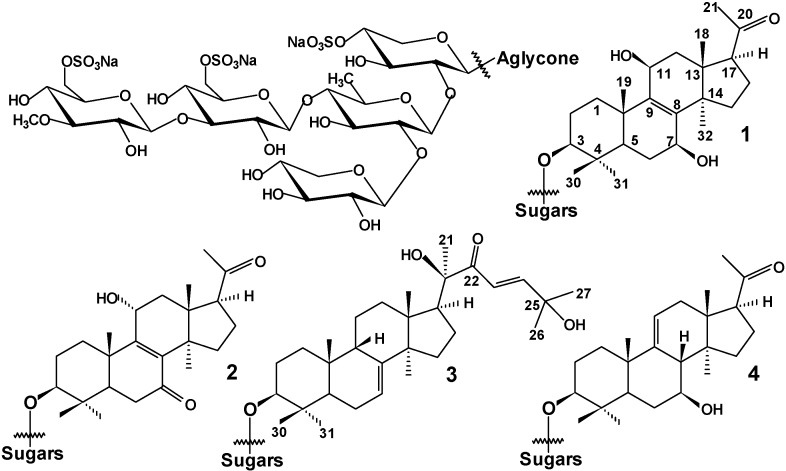
Structures of the glycosides: **1**—fallaxoside D_4_; **2**—fallaxoside D_5_; **3**—fallaxoside D_6_; **4**—fallaxoside D_7_.

The presence five characteristic doublet signals at δ_H_ 4.76–5.22 (1H, d, *J* = 6.9–8.4 Hz), correlated by HSQC spectra with the signals of anomeric carbons at δ_C_ 102.0–105.1 in the ^1^H-NMR spectra of the carbohydrate chains of fallaxosides D_4_ (**1**), D_5_ (**2**), D_6_ (**3**) and D_7_ (**4**) (Scheme 1) and known fallaxosides D_1_ and D_2_ [7] were indicative of a pentasaccharide chain and β-configurations of glycosidic bonds. The positions of all the interglycosidic linkages and the place of linkage of the carbohydrate chain to an aglycone were deduced by the analysis of the ROESY and HMBC spectra of the carbohydrate parts of the glycosides (Table 1). Indeed, the cross-peaks between H-1 of the first monosaccharide residue (xylose) and H-3 (C-3) of an aglycone; H-1 of the second mono- saccharide residue (quinovose) and H-2 (C-2) of the first monosaccaharide residue (xylose); H-1 of the third monosaccharide residue (glucose) and H-4 (C-4) of the second monosaccharide residue (quinovose); H-1 of the fourth monosaccharide residue (3-*O*-methylglucose) and H-3 (C-3) of the third monosaccharide unit (glucose); H-1 of the fifth monosaccharide residue (xylose) and H-2 (C-2) of the second monosaccharide residue (quinovose) were observed. The δ_C_ values characteristic of α- and β-shifting effects of sulfate groups were observed for C-4 (δ_C_ 76.1) and C-5 (δ_C_ 64.0) of the first xylose residue, for C-6 (δ_C_ 67.3) and C-5 (δ_C_ 74.8) of the glucose residue and C-6 (δ_C_ 67.0) and C-5 (δ_C_ 75.5) of terminal 3-*O*-methylglucose residue. These data indicated the presence of a pentasaccharide carbohydrate chain with three sulfate groups.

The ^13^C-NMR spectra of the carbohydrate parts of compounds **1**–**4** (Table 1) were identical to each other and coincided with those of cucumariosides of the group A_7_ isolated first from *Cucumaria japonica* [8] and with the corresponding spectra of fallaxosides D_1_ and D_2_ having the same carbohydrate chain [6]. The structure of such a carbohydrate chain was previously elucidated by desulfation whereby a known desulfated derivative was obtained. This derivative was obtained from a monosulfated glycoside where very detailed chemical evidence of the sugar sequence was obtained by a variety of methods including specific enzymatic hydrolysis, periodate oxidation and Smith degradation, etc. All the obtained progenins were characterized by ^13^C-NMR [8]

The NMR spectra of the aglycone part of fallaxoside D_4_ (**1**) revealed the presence of 24 carbon atoms (Table 2), including in six methylenes, five methines and six methyl groups as well as seven quaternary carbon signals, that corresponded to the 22,23,24,25,26,27-hexanorlanostane type of aglycones previously found in glycosides of *C. fallax* [6]. The resonances of two olefinic carbons with δ_C_ 139.9 (C-8) and 142.9 (C-9) were assigned to the tetrasubstituted double bond. The HMBC correlations between H-32 (δ_H_ 1.00, 3H, s) and C-8 and between H-11 (δ_H_ 4.81, 1H, brd, *J* = 6.8 Hz) and C-9 as well as between H-19 (δ_H_ 1.57, 3H, s) and C-9 confirmed the 8(9)-double bond position. The signal at δ_C_ 211.8 indicated the presence of a keto group. The signals at δ_H_ 2.83 (1H, t, *J* = 8.8 Hz, H-17) and δ_C_ 58.9 (C-17) were assigned to a methine group adjacent to a ketone group. The HMBC correlations from H-17 and H-21 (δ_H_ 2.14, 3H, s) to the carbon at δ_C_ 211.8 (C-20) allowed the positioning of the group at C-20. There were two downshifted resonances in the ^13^C-NMR spectrum of **1** at δ_C_ 67.7 (C-7) and δ_C_ 65.2 (C-11) corresponding to the oxygen bearing allylic type methines suggesting the presence of two hydroxyls. Their positions at C-7 and C-11 were corroborated by the correlations from H-6b (δ_H_ 1.99, 1H, m) to C-7 and from H-12b (δ_H_ 2.27, 1H, d, *J* = 14.2 Hz) to C-11 in the HMBC spectrum as well as ROESY correlations between H-1(2.47 m) and H-11 and between H-5 (1.07 dd, 2.7, 15.6 Hz) and H-7. Seeing that absolute configuration at C-5 with α-orientation of hydrogen, as well as configurations of C-10, C-13, C-14, and C-17 stereocenters in the sea cucumber triterpene glycosides were established earlier [9], the H-5–H-7 H-15α–H7 and H-32–H-7 NOESY correlations are indicative of β-orientation of hydroxyl group at C-7. The configuration of the C-11 stereocenter in **1** was proposed by analysis of the ROESY spectrum and MM2 optimized models of **1**. The β-orientation of hydroxyl group at C-11 was proposed, based on the comparison of MM2 optimized models of aglycones with α- and β-oriented hydroxyls. The observed in the ROESY spectrum of **1** correlations from H-11 to H-1 and *vice versa* would be realized for both 11-α and 11β-hydroxyls. However, the correlations from H-11 with methyl groups H-19 and H-18 should be the realized in the ROESY spectrum at 11α-hydroxy orientation. Nevertheless, these correlations were absent in the ROESY spectrum of **1**. Hence the β-orientation of the hydroxyl group at C-11 is the most probable.

The HR ESI MS (negative and positive ion modes) of fallaxoside D_4_ (**1**) exhibited ion peaks [M_3Na_ − H_2_O − Na]^−^ at *m*/*z* 1403.3543 (calc. 1403.3572), [M_3Na_ − H_2_O − 2Na]^2−^ at *m*/*z* 690.1841 (calc. 690.1840) and [M_3Na_ − H_2_O − 3Na]^3−^ at *m*/*z* 452.4596 (calc. 452.4596). The molecular formula of **1** was deduced as C_53_H_83_O_35_S_3_Na_3_ based on the HR ESI MS and ^13^C-NMR spectroscopic data. However, the ions observed in the mass spectra corresponded to the loss of water upon ionization of fallaxoside D_4_ (**1**), presumably due to the lability of the dihydroxy-ene-fragment in the rings B and C of its aglycone. The structure of the carbohydrate chain of **1** was also confirmed by fragment ion peaks at *m*/*z* 929, corresponding to a monodesulfated carbohydrate chain [MeGlcOSO_3_Na + GlcOSO_3_Na + Qui + Xyl + XylOSO_3_Na − Na_2_SO_4_]^−^ or [M_3Na_ − Na_2_SO_4_ − agl (C_24_H_37_O_3_)]^−^. Other fragment ions at *m*/*z* 827.2 corresponded to a didesulfated carbohydrate chain [MeGlcOSO_3_Na + GlcOSO_3_Na + Qui + Xyl + XylOSO_3_Na − Na_2_SO_4_ − NaSO_3_ + H]^−^ or [M_3Na_ − Na_2_SO_4_ − NaSO_3_ − agl (C_24_H_37_O_3_) + H]^−^; at *m*/*z* 797.1 [M_3Na_ − Na_2_SO_4_ − agl (C_24_H_37_O_3_) − Xyl + H]^−^; at *m*/*z* 695.2 [M_3Na_ − Na_2_SO_4_ − NaSO_3_ − agl (C_24_H_37_O_3_) − Xyl + 2H]^−^; at *m*/*z* 563.1 [M_3Na_ − Na_2_SO_4_ − NaSO_3_ − agl (C_24_H_37_O_3_) − 2Xyl + 3H]^−^; at *m*/*z* 519 [M_3Na_ − Na_2_SO_4_ − agl (C_24_H_37_O_3_) − 2Xyl − Qui + H]^−^; at *m*/*z* 417.1 [M_3Na_ − Na_2_SO_4_ − NaSO_3_ − agl (C_24_H_37_O_3_) − 2Xyl − Qui + 2H]^−^, observed in the ESI MS/MS spectra of **1**.

Taken together all these data indicate that fallaxoside D_4_ (**1**) is 22,23,24,25,26,27-hexanor-3β-*O*-{6-*O*-sodium sulfate-3-*O*-methyl-β-d-glucopyranosyl-(1→3)-6-*O*-sodium sulfate-β-d-glucopyranosyl-(1→4)-[β-d-xylopyranosyl-(1→2)]-β-d-quinovopyranosyl-(1→2)-4-*O*-sodium sulfate-β-d-xylopyranosyl}-7β,11β-dihydroxy-20-ketolanost-8-ene.

The structure of the aglycone moiety of fallaxoside D_5_ (**2**) was proved to be similar and NMR spectra also confirmed the 22,23,24,25,26,27-hexanorlanostane skeleton system of its aglycone. The difference in the spectra (Table 3) was in the presence of an additional signal of a ketone group at δ_C_ 201.4 instead of one of hydroxyl group signals in the ^13^C-NMR spectrum. 

The signals of the quaternary olefinic carbons indicated the presence of 8(9)-double bond and were downshifted (δ_C_ 140.0 and 163.1) when compared with those in the ^13^C-NMR spectrum of **1**, suggesting that the ketone group adjoins the double bond. Additionally, the signals at δ_H_ 2.56 (1H, m, H-6a) and δ_H_ 2.50 (1H, m, H-6b) demonstrated the formation of a spin coupled system in the COSY spectrum with the signal of H-5 (1H, dd, *J* = 3.9, 13.5 Hz) only and were also noticeably downshifted. These data indicated the closeness of these atoms to the ketone group that was positioned at C-7. The HMBC correlation from both H_2_-6 to C-7 (δ_C_ 201.4) and C-10 (δ_C_ 40.6) confirmed the ketone position. Hence, the hydroxyl group at C-7 of the aglycone of **1** was substituted with a ketone group in the aglycone of **2**. The signal at δ_C_ 64.1 (C-11) in the ^13^C-NMR spectrum of **2**, correlated by the HSQC with the resonance at δ_H_ 4.70 (1H, dd, *J* = 5.4, 8.9 Hz, H-11) indicated the presence of oxygen bearing methine group. The HMBC correlations from both H-12 (δ_H_ 2.58, 1H, dd *J* = 8.8, 13.1 Hz, H-12a) and (δ_H_ 2.40, 1H, dd *J* = 5.1, 13.4 Hz, H-12b) to C-11 corroborated the attachment of hydroxyl group to C-11.

The orientation of hydroxyl group was proposed as α based on the ROESY spectrum. Clear NOE correlations from H-11 to both β-oriented methyl groups Me-18 and Me-19 were observed. Hence the hydroxyl groups at C-11 are opposite oriented in fallaxosides D_4_ (**1**) and D_5_ (**2**) when compared each other. The HRESIMS (negative ion mode) of fallaxoside D_5_ (**2**) exhibited quasi-molecular ion peaks at *m*/*z* 1419.3502 (calc. 1419.3521) [M_3Na_ − Na]^−^, at *m*/*z* 698.1806 (calc. 698.1815) [M_3Na_ − 2Na]^2−^ and at *m*/*z* 457.7911 (calc. 457.7912) [M_3Na_ − 3Na]^3−^. This and ^13^C-NMR spectroscopic data allowed the determination of the molecular formula of **2** as C_53_H_81_O_35_S_3_Na_3_. The presence of sulfate groups and sequence of monosaccharide units in the carbohydrate chain of **2** was confirmed by fragmentary ions at *m*/*z* 1299.4 [M_3Na_ − Na − HSO_4_Na]^−^, 1179.4 [M_3Na_ − Na − 2HSO_4_Na]^−^, 911.1 [M_3Na_ − Na_2_SO_4_ − agl + H]^−^; 877.3 [M_3Na_ − Na − MeGlcOSO_3_Na − GlcOSO_3_Na +H]^−^; 797.1 [M_3Na_ − Na_2_SO_4_ − agl − Xyl + H]^−^; 745.3 [M_3Na_ − Na − MeGlcOSO_3_Na − GlcOSO_3_Na − Xyl + 2H]^−^; 665.1 [M_3Na_ − Na_2_SO_4_ − agl − 2Xyl + 2H]^−^; 599.3 [M_3Na_ − Na − MeGlcOSO_3_Na − GlcOSO_3_Na − Qui − Xyl + H]^−^; 519 [M_3Na_ − Na_2_SO_4_ − agl − 2Xyl − Qui + H]^−^ detected in the ESI MS/MS (negative ion mode) spectrum. All these data indicate that fallaxoside D_5_ (**2**) is 22,23,24,25,26, 27-hexanor-3β-*O*-{6-*O*-sodium sulfate-3-*O*-methyl-β-d-glucopyranosyl-(1→3)-6-*O*-sodium sulfate-β-d-glucopyranosyl-(1→4)-[β-d-xylopyranosyl-(1→2)]-β-d-quinovopyranosyl-(1→2)-4-*O*-sodium sulfate-β-d-xylopyranosyl}-11α-hydroxy-7,20-diketolanost-8-ene.

The ^13^C-NMR spectrum of the aglycone part of fallaxoside D_6_ (**3**) revealed the presence of 30 carbons indicating the presence of an aglycone with normal non-shortened side chain (Table 4). The absence of a γ-lactone was deduced from the absence of the signal at ≈ δ_C_ 176 and contemporary presence of the resonances of Me-18 (δ_C_ 24.8, C-18, δ_H_ 1.28, 3H, s, H-18) in the ^13^C- and ^1^H-NMR spectra, respectively, indicating the aglycone of fallaxoside D_6_ (**3**) to be of the lanostane type without a lactone, in contrast with aglycones of the majority of sea cucumber glycosides. The resonances of an olefinic methine group at δ_C_ 122.2 (C-7) and δ_H_ 5.59 (1H, m, H-7) in NMR spectra as well as of an olefinic quaternary carbon at δ_C_ 149.7 (C-8) in the ^13^C-NMR spectrum were assigned to the 7(8)-double bond typical of many sea cucumber glycosides [1,9]. The presence of an isolated H-5/H_2_-6/H-7/H-9/H-11/H-12 spin system in the COSY spectrum of **3** as well as the HMBC correlation between H-32 (δ_H_ 1.03, 3H, s) and C-8 confirmed the double bond position. The signal at δ_C_ 205.3 and resonances of two olefinic tertiary carbons at δ_C_ 119.9 and 156.5 in the ^13^C-NMR spectrum indicated the presence of a keto group and an additional double bond, correspondingly. Extensive analysis of the COSY, HSQC and HMBC spectra of **3** revealed these functionalities could be positioned only in the side chain of the aglycone. Actually the multiplicity and the coupling constants of olefinic protons at δ_H_ 7.41 (1H, d, *J* = 15.5 Hz, H-23) and δ_H_ 7.40 (1H, d, *J* = 15.5 Hz, H-24) were indicative of a *Z*-configured 23(24)-double bond vicinal on the one hand to the keto group (C-22) and from the other hand to the quaternary oxygen bearing carbon at δ_C_ 70.5 (C-25). The presence of the resonances of two heminal methyl groups at δ_H_1.47 (3H, s, H-26) and δ_H_ 1.48 (3H, s, H-27) in the ^1^H-NMR spectrum correlated by HSQC spectrum with corresponding carbons resonances at δ_C_ 29.3 (C-26) and δ_C_ 29.2 (C-27) as well as the HMBC correlations from H-26 and H-27 to C-24 and C-25 and from H-23 and H-24 to C-22 and C-25 confirmed the structure of side chain having 22-keto- and 25-hydroxyl groups and 23*Z*,24-double bond. The correlations observed in the ROESY spectrum of **3** (Table 4) corroborated the configurations of stereocenters C-3, C-5, C-10, C-13, C-14, C-17 as well as (20*R*) configuration (NOE between H-16 and H-23 and vice versa) established earlier for similar aglycone of frondoside C [10]. A ROESY correlations of a signal at 2.28 brd, *J* = 12.8 Hz, H-9 with signals of methyl group at C-10 and C-14 established the rare 9β-H configuration in **3** characteristic of 7(8)-unsaturated sea cucumber glycosides [1,9,11]. Thus, these data allowed us to determine all the structural and stereochemical features of this unusual aglycone.

The HRESIMS (negative ion mode) of fallaxoside D_6_ (**3**) exhibited pseudomolecular ion peaks [M_3Na_ − 2Na]^2−^ at *m*/*z* 740.2279 (calc. 740.2284) and [M_3Na_ − 3Na]^3−^ at *m*/*z* 485.8224 (calc. 485.8225). This and ^13^C-NMR spectroscopic data allowed the determination of the molecular formula of **3** as C_59_H_93_O_35_S_3_Na_3_. The presence of sulfate groups and sequence of monosaccharide units in the carbohydrate chain of **3** was confirmed by the fragmentary ion peaks at *m*/*z* 1383.4 [M_3Na_ − NaHSO_4_]^−^; 827.2 [M_3Na_ − Na_2_SO_4_ − NaSO_3_ − agl + H]^−^; 797.1 [M_3Na_ − Na_2_SO_4_ − agl − Xyl + H]^−^; 695.2 [M_3Na_ − Na_2_SO_4_ − NaSO_3_ − agl − Xyl + 2H]^−^; 563.1 [M_3Na_ − Na_2_SO_4_ − NaSO_3_ − agl − 2Xyl + 3H]^−^; 519 [M_3Na_ − Na_2_SO_4_ − agl − 2Xyl − Qui + H]^−^ detected in the ESI MS/MS (negative ion mode) spectrum.

All these data indicate that fallaxoside D_6_ (**3**) is 3β-*O*-{6-*O*-sodium sulfate-3-*O*-methyl-β-d-glucopyranosyl-(1→3)-6-*O*-sodium sulfate-β-d-glucopyranosyl-(1→4)-[β-d-xylo-pyranosyl-(1→2)]-β-d-quinovopyranosyl-(1→2)-4-*O*-sodium sulfate-β-d-xylopyranosyl}-22-keto-25-hydroxylanosta-7,23*Z*-diene.

The structure of the aglycone moiety of fallaxoside D_7_ (**4**) was found by extensive NMR spectroscopy (Table 5) to be similar to that of **1**–**2**, indicating the presence of a 22,23,24,25,26,27-hexanorlanostane aglycone with the keto group at C-20 (δ_C_ 212.5). The double bond was positioned as 9(11) according to the signal of the quaternary olefinic carbon at δ_C_ 147.6 (C-9) and olefinic methine resonances at δ_C_ 116.0 (C-11) in the ^13^C-NMR spectrum and δ_H_ 5.27 (1H, m, H-11) in the ^1^H-NMR spectrum. The long range COSY correlation H-8/H-11 as well as the characteristic correlation from Me-19 (δ_H_ 1.00, 3H, s, H-19) to C-9 confirmed the double bond position. The signal at δ_C_ 71.6 (C-7) in the ^13^C-NMR spectrum of **4**, correlated by the HSQC with the resonance at δ_H_ 3.83 (1H, m, H-7) indicated the attachment of hydroxyl group to this carbon. Its position as C-7 was confirmed by the COSY spectrum, where the signals of spin coupled system H-5/H_2_-6/H-7/H-8 were observed.

The orientation of hydroxyl group was proposed as β based on the ROESY spectrum. Clear NOE correlations from H-7 to both α-oriented H-5 (δ_H_ 0.86, 1H, brd, *J* = 13.7 Hz) and Me-32 (δ_H_ 0.99, 3H, s) and vice versa were observed. (8*S*)-Configuration was proposed based on the correlations between H-8 and the both Me-18 and Me-19, observed in the ROESY spectrum of **4**.

The HR ESI MS (negative ion mode) of fallaxoside D_7_ (**4**) exhibited ion peaks [M_3Na_ − 2Na]^2−^ at *m*/*z* 691.1917 (calc. 691.1918) and [M_3Na_ − 3Na]^3−^ at *m*/*z* 453.1320 (calc. 453.1315). This and ^13^C-NMR spectroscopic data allowed the determination of the molecular formula of **4** as C_53_H_83_O_34_S_3_Na_3_. The identity of the carbohydrate chain structure of fallaxoside D_7_ (**4**) to those of **1**–**3** confirmed by the presence of the fragmentary ion peaks with the same *m*/*z*: 797.1, 695.2, 665.1, 563.1, 519.0 and 417.1 in the ESI MS/MS (negative ion mode) spectrum of **4**.

On the base of all the above discussed data, the structure of fallaxoside D_7_ (**4**) was established to be 22,23,24,25,26,27-hexanor-3β-*O*-{6-*O*-sodium sulfate-3-*O*-methyl-β-d-glucopyranosyl-(1→3)-6-*O*-sodium sulfate-β-d-glucopyranosyl-(1→4)-[-β-d-xylopyranosyl-(1→2)]-β-d-quinovopyranosyl-(1→2)-4-*O*-sodium sulfate-β-d-xylopyranosyl}-7β-hydroxy-20-ketolanost-9(11)-ene.

The cytotoxic activities of fallaxosides D_4_ (**1**), D_5_ (**2**), D_6_ (**3**) and D_7_ (**4**) against mouse spleen lymphocytes and ascite form of mouse Ehrlich carcinoma cells along with hemolytic activity against mouse erythrocytes were studied. None of these glycosides were active in these tests at the dosage studied (IC_50_ > 100 µM/mL). This could be explained by the absence of the 18(20)-lactone moiety that is essential for the membranolytic action of sea cucumber glycosides and by the presence of three sulfate groups that decreased the membranolytic activity of the glycosides [12]. 

It is most probable that holostane type glycosides play a role in chemical defense against predators because of their strong membranolytic activities and not only as regulators of oocyte maturation. Non-holostane glycosides may play only a regulatory role and are evolutionary precursors of holostane glycosides [12].

In summary, four novel triterpene glycosides **1**–**4** have been isolated from the sea cucumber *Cucumaria fallax* along with the series of other uncommon glycosides [6]. All of these compounds contain new non-holostane aglycones with some unprecedented structural features. It is obvious that the isolated glycosides, including the earlier studied fallaxoside C_2_ and D_2_ (having a 7-keto-8(9)-en fragment), C_1_ and D_1_ (having a 7,11-diketo-8(9)-en fragment) [6], D_4_ (**1**) (with a 7,11-dihydroxy-8(9)-en fragment), D_5_ (**2**) (with a 7-keto-11-hydroxy-8(9)-en fragment) and D_7_ (**4**) (with a 7-hydroxy-9(11)-en fragment), are biogenetically related with each other. They have oxygen-containing functional groups (ketones or hydroxyls) at the same C-7 and/or C-7 and C-11 positions of aglycones. Another unusual structural feature of many isolated glycosides includes the presence of a 8(9)-double bond in the majority of glycosides isolated from this species. This suggests that in *C. fallax*, the protosterol cation, formed from squalene, is cyclized not only into 9(11)-unsaturated parkeol or into 9β-*H*-lanosta-7,24-dienol, as shown earlier in other sea cucumbers [13], but also into lanosta-8(9),24-diene-3β-ol (lanosterol).

## 3. Experimental Section

### 3.1. General Experimental Procedures

Melting points were determined with a Kofler-Thermogenerate apparatus (Leica VMTG, Wien, Austria). Specific rotation was measured on a 343 Polarimeter (Perkin-Elmer Corporation, Waltham, MA, USA). NMR spectra were recorded on an Advance III-700 spectrometer (Bruker Bio Spin, Rheinstetten, Germany) at 700.13 MHz/176.04 MHz (^1^H/^13^C) in C_5_D_5_N/D_2_O (4/1) with TMS as an internal reference (δ = 0). The ESIMS (negative ion mode) were recorded using an Agilent 6510 Q-TOF apparatus (Agilent Corporation, Palo Alto, CA, USA), 50% MeOH was used as the solvent, sample concentration 0.01 mg/mL. HPLC was performed using an Agilent 1100 chromatograph equipped with a differential refractometer on a Ascentis RP Amide (10 × 250 mm, 5 µm) column (Supelco, Bellefonte, PA, USA).

### 3.2. Animal Material

The samples of *Cucumaria fallax* (Cucumariidae, Dendrochirotida) were collected during the 41-st scientific cruise of the research vessel *Akademik Oparin* in the Pacific Ocean near Black Brothers Islands, Kurile Islands (46°23′9′′ N, 150°46′25′′ E) on 20 July, 2011 using Sigsbee trawl from the depth of 150 m (collector Kirill Minin, P.P. Shyrshov Institute of Oceanology of the Russian Academy of Sciences, Moscow, Russia). The sea cucumber taxonomic identification was carried out by Dr. Vadim G. Stepanov (Kamchatka Department of the Institute of Geography, Far East Division of the Russian Academy of Sciences, Petropavlosk-Kamchatsky, Russia), the voucher specimen is deposited in collection of Kamchatka Department of the Institute of Geography.

### 3.3. Extraction and Isolation

The sea cucumbers were minced and extracted with 70% ethanol under reflux during 5 h. The concentrated in vacuo ethanolic extract of *C. fallax* (28.2 g of dry wt after ethanol extraction) was sequentially submitted to column chromatography on Polychrom-1 (powdered Teflon) in H_2_O→50% ethanol and on Si gel using CHCl_3_/EtOH/H_2_O (100:125:25 and 100:150:50) as mobile phases to obtain the fraction A_7_ (165 mg). Further separation of the fraction by HPLC on a semi-preparative Supelco Ascentis RP-Amide (10 × 250 mm) reverse phase column using MeOH/H_2_O/NH_4_OAc (1 M water solution) as mobile phase in ratio 60/39/1 gave the subfractions A_7_I–A_7_V, each of them was subsequently rechromatographed. The HPLC of subfraction A_7_I with the same solvent system in ratio of 35/64/1 gave 3.5 mg of fallaxoside D_4_ (**1**) and 3.5 mg of fallaxoside D_5_ (**2**). The HPLC of subfraction A_7_II using the solvent system in ratio of 50/49/1 followed by 45/54/1 and 47/51/2 gave 1.8 mg of fallaxoside D_7_ (**4**). The HPLC of subfraction A_7_V with the same solvents in ratio of 58/41/1 gave 7.2 mg of fallaxoside D_6_ (**3**).

*Fallaxoside D_4_* (**1**): Colorless powder; MP: 212–214 °C; ${\left[\alpha \right]}_{D}^{20}$ −2 (*c* 0.1, 50% MeOH); NMR: See Table 1 and Table 2; HRESIMS (−) *m*/*z* 1403.3543 (calc. for C_53_H_83_O_35_S_3_Na_3_, 1403.3572) [M_3Na_ − H_2_O − Na]^−^, *m*/*z* 690.1841 (calc. 690.1840) [M_3Na_ − H_2_O − 2Na]^2−^, 452.4596 (calc. 452.4596) [M_3Na_ − H_2_O − 3Na]^3−^; ESI MS/MS (−) *m*/*z* 929 [M_3Na_ − Na_2_SO_4_ − agl (C_24_H_37_O_3_)]^−^; 827.2 [M_3Na_ − Na_2_SO_4_ − NaSO_3_ − agl (C_24_H_37_O_3_) + H]^−^; 797.1 [M_3Na_ − Na_2_SO_4_ − agl (C_24_H_37_O_3_) − Xyl + H]^−^; 695.2 [M_3Na_ − Na_2_SO_4_ − NaSO_3_ − agl (C_24_H_37_O_3_) − Xyl + 2H]^−^; 563.1 [M_3Na_ − Na_2_SO_4_ − NaSO_3_ − agl (C_24_H_37_O_3_) − 2Xyl + 3H]^−^; 519 [M_3Na_ − Na_2_SO_4_ − agl (C_24_H_37_O_3_) − 2Xyl − Qui + H]^−^; 417.1 [M_3Na_ − Na_2_SO_4_ − NaSO_3_ − agl (C_24_H_37_O_3_) − 2Xyl − Qui + 2H]^−^.

*Fallaxoside D_5_* (**2**): Colorless powder; MP: 206–208 °C; ${\left[\alpha \right]}_{D}^{20}$ –3 (*c* 0.1, 50% MeOH); NMR: see Table 1 and Table 3; HRESIMS (−) *m*/*z* 1419.3502 (calc. for C_53_H_81_O_35_S_3_Na_3_, 1419.3521) [M_3Na_ − Na]^−^, 698.1806 (calc. 698.1815) [M_3Na_ − 2Na]^2−^, 457.7911 (calc. 457.7912) [M_3Na_ − 3Na]^3−^; ESI MS/MS (−) *m*/*z* 1299.4 [M_3Na_ − Na − HSO_4_Na]^−^, 1179.4 [M_3Na_ − Na − 2HSO_4_Na]^−^, 911.1 [M_3Na_ − Na_2_SO_4_ − agl + H]^−^; 877.3 [M_3Na_ − Na − MeGlcOSO_3_Na − GlcOSO_3_Na +H]^−^; 797.1 [M_3Na_ − Na_2_SO_4_ − agl − Xyl + H]^−^; 745.3 [M_3Na_ − Na − MeGlcOSO_3_Na − GlcOSO_3_Na − Xyl + 2H]^−^; 665.1 [M_3Na_ − Na_2_SO_4_ − agl − 2Xyl + 2H]^−^; 599.3 [M_3Na_ − Na − MeGlcOSO_3_Na − GlcOSO_3_Na − Qui − Xyl + H]^−^; 519 [M_3Na_ − Na_2_SO_4_ − agl − 2Xyl − Qui + H]^−^.

*Fallaxoside D_6_* (**3**): Colorless powder; MP: 225–227 °C; ${\left[\alpha \right]}_{D}^{20}$ –8 (*c* 0.1, 50% MeOH); NMR: see Table 1 and Table 4; HRESIMS (−) *m*/*z* 740.2279 (calc. for C_59_H_93_O_35_S_3_Na_3_, 740.2284) [M_3Na_ − 2Na]^2−^, 485.8224 (calc. 485.8225) [M_3Na_ − 3Na]^3−^; ESI MS/MS (−) *m*/*z* 1383.4 [M_3Na_ − NaHSO_4_]^−^; 827.2 [M_3Na_ − Na_2_SO_4_ − NaSO_3_ − agl + H]^−^; 797.1 [M_3Na_ − Na_2_SO_4_ − agl − Xyl + H]^−^; 695.2 [M_3Na_ − Na_2_SO_4_ − NaSO_3_ − agl − Xyl + 2H]^−^; 563.1 [M_3Na_ − Na_2_SO_4_ − NaSO_3_ − agl − 2Xyl + 3H]^−^; 519 [M_3Na_ − Na_2_SO_4_ − agl − 2Xyl − Qui + H]^−^.

*Fallaxoside D_7_* (**4**): Colorless powder; MP: 210–212 °C; ${\left[\alpha \right]}_{D}^{20}$ –5 (*c* 0.1, 50% MeOH); NMR: See Table 1 and Table 5; HRESIMS (−) *m*/*z* 691.1917 (calc. for C_53_H_83_O_34_S_3_Na_3_, 691.1918) [M_3Na_ − 2Na]^2−^, 453.1320 (calc. 453.1315) [M_3Na_ − 3Na]^3−^; ESI MS/MS (−) *m*/*z* 797.1 [M_3Na_ − Na_2_SO_4_ − agl − Xyl + H]^−^; 695.2 [M_3Na_ − Na_2_SO_4_ − NaSO_3_ − agl − Xyl + 2H]^−^; 665.1 [M_3Na_ − Na_2_SO_4_ − agl − 2Xyl + 2H]^−^; 563.1 [M_3Na_ − Na_2_SO_4_ − NaSO_3_ − agl − 2Xyl + 3H]^−^, 519.0 [M_3Na_ − Na_2_SO_4_ − agl − 2Xyl − Qui + H]^−^; 417.1 [M_3Na_ − Na_2_SO_4_ − NaSO_3_ − agl − 2Xyl − Qui + 2H]^−^.

### 3.4. Cell Culture

Splenocytes from CD-1 line mice were used. The spleen were isolated from mice and centrifuged (450× *g*) for 5 min. The splenocytes were washed three times and resuspended in phosphate-buffered saline, pH 7.2–7.4 at final concentration 2–5 × 10^6^ cells/mL.

### 3.5. Cytotoxicity Activity

Water solutions (10 µL), containing different concentrations (0.12–100.00 µM) of the tested substances and 90 µL of the cell suspension were added to the wells of a 96-well plate and incubated for 1 h at 37 °C. After the incubation, 10 µL of the cell suspension were added to 10 µL of trypan blue solution (0.4% in PBS) and placed on a slide. After 1–5 min incubation the amount of alive and dead cells were calculated with an Imager A1 optical microscope (Carl Zeiss, Oberkochen, Germany) using the AxioVision (Carl Zeiss) 4.7.1 software. The cytotoxic activity of the substances was calculated as the ratio of the dead cells to general cells amount. ED_50_ was calculated with SigmaPlot 10.0 software.

### 3.6. Hemolytic Activity

Blood was taken from a CD-1 mouse. The erythrocytes were washed three times with 0.9% NaCl, centrifuged (450× *g*) on a LABOFUGE 400R centrifuge (Heraeus, Hanau, Germany) for 5 min [14] followed by resuspension in phosphate-buffered saline (PBS), pH 7.2–7.4. Erythrocytes were used at a concentration providing an optical density of 1.5 at 700 nm for a non-hemolyzed sample. Twenty µL of a water solution of test substance with a fixed concentration (0.12–100.00 µM) were added to a well of a 96-well plate containing 180 mL of the erythrocyte suspension and incubated for 1 h at 37 °C. The plates were centrifuged (900× *g*) on a LMC-3000 laboratory centrifuge (Biosan, Riga, Latvia) for 10 min as proposed in [15]. Ten µL of the supernatant were placed on special microplate with a plate bottom for determination of the optical density on a Multiskan FC spectrophotometer (Termo Fisher Scientific, Waltham, MA, USA) at λ = 570 nm. ED_50_ values were calculated using the SigmaPlot 3.0 software. Triton X-100 (Biolot, Saint Petersburg, Russian Federation) at a concentration of 1%, causing 100% cell hemolysis was used as positive control. The erythrocyte suspension in phosphate-buffered saline, pH 7.2–7.4 (PBS) with 20 µL of the solvent without a tested compound was used as negative control.

## Figures and Tables

**Table 1:** NMR Spectroscopic data (700 MHz, C_5_D_5_N/D_2_O (4/1 *v*/*v*)) of the carbohydrate moieties of **1**–**4**.

Atom	δ_C_ mult. ^a,b,c^	δ_H_ mult. (*J* in Hz) ^d^	HMBC	ROESY
Xyl1 (1→C-3)				
1	104.7 CH	4.76 d (6.9)	C: 3	H-3, H-3, 5 Xyl1
2	**81.3** CH	3.97 t (8.6)	C: 1 Qui2; C: 1, 3 Xyl1	H-1 Qui2; H-4 Xyl1
3	75.2 CH	4.31 t (8.6)	C: 2, 4 Xyl1	H-1, 5 Xyl1
4	*76.1* CH	4.98 dd (8.6, 13.8)	C: 3 Xyl1	H-2 Xyl1
5	64.0 CH_2_	4.76 d (11.2)	C: 1, 3 Xyl1	
		3.87 dd (8.6, 11.2)		H-1, 3 Xyl1
Qui2 (1→2Xyl1)				
1	102.0 CH	5.20 d (7.8)	C: 2 Xyl1	H-2 Xyl1; H-3, 5 Qui2
2	82.4 CH	3.92 t (8.6)	C: 1 Xyl5; C: 1, 3 Qui2	H-1 Xyl5; H-4 Qui2
3	75.2 CH	3.98 t (8.6)	C: 2, 4 Qui2	H-5 Qui2
4	**86.3** CH	3.43 t (8.6)	C: 1 Glc3; C: 3, 5, 6 Qui2	H-1 Glc3; H-2, 6 Qui2
5	70.8 CH	3.56 dd (6.0, 9.5)		H-1, 3, 6 Qui2
6	17.8 CH_3_	1.55 d (6.0)	C: 4, 5 Qui2	H-4, 5 Qui2
Glc3 (1→4Qui2)				
1	103.9 CH	4.78 d (7.8)	C: 4 Qui2	H-4 Qui2; H-5 Glc3
2	73.4 CH	3.81 m	C: 1, 3 Glc3	
3	**86.5** CH	4.13 t (8.6)	C: 1 MeGlc4; C: 2, 4 Glc3	H-1 MeGlc4; H-1 Glc3
4	69.1 CH	3.80 t (8.6)	C: 5, 6 Glc3	
5	74.8 CH	4.08 m		H-1 Glc3
6	*67.3* CH_2_	4.94 d (11.2)		
		4.59 dd (6.9, 11.2)	C: 5 Glc3	H-4 Glc3
MeGlc4 (1→3Glc3)				
1	104.7 CH	5.15 d (8.4)	C: 3 Glc3	H-3 Glc3; H-3, 5 MeGlc4
2	74.3 CH	3.78 t (8.4)	C: 1 MeGlc4	H-4 MeGlc4
3	86.3 CH	3.64 t (9.3)	C: 2, 4 MeGlc4, OMe	H-1, 5 MeGlc4
4	69.8 CH	4.01 m	C: 3, 5 MeGlc4	H-2, 6 MeGlc4
5	75.5 CH	4.01 m	C: 4, 6 MeGlc4	H-1, 3 MeGlc4
6	*67.0* CH_2_	4.92 brd (11.0)	C: 4, 5 MeGlc4	
		4.75 brd (8.4)	C: 5 MeGlc4	
OMe	60.4 CH_3_	3.80 s	C: 3 MeGlc4	
Xyl5 (1→2Qui2)				
1	105.1 CH	5.22 d (7.6)	C: 2 Qui2	H-2 Qui2; H-3, 5 Xyl5
2	74.8 CH	3.91 t (7.6)	C: 1, 3 Xyl5	
3	76.3 CH	4.07 t (8.4)	C: 2, 4 Xyl5	H-1, 5 Xyl5
4	70.1 CH	4.05 m	C: 3 Xyl5	H-2 Xyl5
5	66.4 CH_2_	4.28 dd (5.1, 11.8)	C: 1, 4 Xyl5	
		3.66 t (9.3)	C: 1, 3, 4 Xyl5	H-1, 3 Xyl5

^a^ Recorded at 176.04 MHz; ^b^ Bold = interglycosidic positions; ^c^ Italic = sulfate position; ^d^ Multiplicity by 1D TOCSY.

**Table 2:** NMR Spectroscopic data (700 MHz, C_5_D_5_N/D_2_O (4/1 *v*/*v*)) of the aglycone moiety of fallaxoside D_4_ (**1**).

Position	δ_C_ Mult. ^a^	δ_H_ Mult. (*J* in Hz)	HMBC	ROESY
1	34.9 CH_2_	2.47 m		H-11, H-19
		1.32 m	C: 18	H-3, H-5, H-11
2	26.6 CH_2_	1.90 m		
		1.79 m		H-19, H-30
3	88.6 CH	3.13 dd (4.7, 12.2)	C: 30, 31, C: 1 Xyl1	H-1, H-5, H-31, H-1 Xyl1
4	39.5 C			
5	51.4 CH	1.07 dd (2.7, 15.6)	C: 4, 19, 30	H-1, H-3, H-7, H-31
6	30.8 CH_2_	2.35 m		H-31
		1.99 m	C: 5, 7, 10	H-19, H-30
7	67.7 CH	4.55 t (8.1)	C: 8, 9	H-5, H-15, H-32
8	139.9 C			
9	142.9 C			
10	38.3 C			
11	65.2 CH	4.81 brd (6.8)	C: 8, 9, 13	H-1
12	42.8 CH_2_	2.53 dd (7.5, 14.2)	C: 13, 18	H-17, H-32
		2.27 d (14.2)	C: 9, 11, 13, 14, 18	H-18, H-21
13	45.2 C			
14	51.5 C			
15	32.0 CH_2_	2.71 brd (10.8) β	C: 14, 32	H-18
		1.60 brt (10.8) α		H-7, H-32
16	22.6 CH_2_	2.41 m		H-18
		1.68 m		H-32
17	58.9 CH	2.83 t (8.8)	C: 12, 13, 16, 18, 20, 21	H-12, H-21, H-32
18	19.7 CH_3_	1.23 s	C: 12, 13, 14, 17	H-12, H-15, H-16, H-19, H-21
19	22.2 CH_3_	1.57 s	C: 1, 5, 9, 10	H-1, H-2, H-5, H-6, H-18, H-30
20	211.8 C			
21	31.3 CH_3_	2.14 s	C: 17, 20	
30	16.4 CH_3_	0.99 s	C: 3, 4, 5, 31	H-2, H-6, H-19, H-31
31	27.9 CH_3_	1.15 s	C: 3, 4, 5, 30	H-3, H-5, H-6, H-30
32	26.1 CH_3_	1.00 s	C: 8, 13, 14, 15	H-7, H-12, H-15, H-16, H-17

^a^ Recorded at 176.04 MHz.

**Table 3:** NMR Spectroscopic data (700 MHz, C_5_D_5_N/D_2_O (4/1 *v*/*v*)) of the aglycone moiety of fallaxoside D_5_ (**2**).

Position	δ_C_ Mult. ^a^	δ_H_ Mult. (*J* in Hz)	HMBC	ROESY
1	34.1 CH_2_	2.29 m		H-3, H-5
		2.01 m	C: 10	H-11
2	26.5 CH_2_	2.00 m	C: 3	H-19
		1.77 m		H-19, H-30
3	87.8 CH	3.13 dd (4.0; 11.6)	C: 4, 30, C: 1 Xyl1	H-1, H-5, H-31, H-1 Xyl1
4	39.5 C			
5	50.5 CH	1.83 dd (3.9; 13.5)	C: 4, 6, 10, 19, 30, 31	H-1, H-3, H-31
6	36.9 CH_2_	2.56 m	C: 5, 7, 10	
		2.50 m	C: 5, 7, 10	H-19, H-30
7	201.4 C			
8	140.0 C			
9	163.1 C			
10	40.6 C			
11	64.1 CH	4.70 dd (5.4; 8.9)	C: 8, 9, 10, 13	H-1, H-18, H-19
12	43.5 CH_2_	2.58 dd (8.8; 13.1)	C: 9, 11, 13, 18	H-18, H-21
		2.40 dd (5.1; 13.4)	C: 11, 13, 17, 18	H-17, H-32
13	48.5 C			
14	48.7 C			
15	33.4 CH_2_	2.30 m	C: 14, 17, 32	H-32
		1.86 m		H-18
16	22.1 CH_2_	2.27 m		H-18
		1.68 m		H-32
17	58.6 CH	2.91 t (9.0)	C: 12, 13, 16, 18, 20	H-12, H-21, H-32
18	18.6 CH_3_	0.58 s	C: 12, 13, 14, 17	H-12, H-15, H-16, H-19, H-21
19	19.8 CH_3_	1.10 s	C: 1, 5, 9, 10	H-2, H-6, H-18
20	211.7 C			
21	31.2 CH_3_	2.15 s	C: 17, 20	H-17, H-18
30	16.0 CH_3_	1.01 s	C: 3, 4, 5, 31	H-2, H-6
31	27.0 CH_3_	1.05 s	C: 3, 4, 5, 30	H-3, H-5, H-6
32	25.0 CH_3_	1.40 s	C: 8, 13, 14, 15	H-12, H-15, H-17

^a^ Recorded at 176.04 MHz.

**Table 4:** NMR spectrosopic data (700 MHz, C_5_D_5_N/D_2_O (4/1 *v*/*v*)) of the aglycone moiety of fallaxoside D_6_ (**3**).

Position	δ_C_ Mult. ^a^	δ_H_ Mult. (*J* in Hz)	HMBC	ROESY
1	35.5 CH_2_	1.32 m		H-11
		1.27 m		
2	27.0 CH_2_	1.95 m		
		1.78 m		H-19, H-30
3	88.9 CH	3.14 dd (4.1, 11.6)	C: 30, C: 1 Xyl1	H-5, H-31, H-1 Xyl1
4	39.5 C			
5	49.7 CH	0.84 brd (12.7)	C: 1, 4, 30	H-1, H-3, H-31
6	23.1 CH_2_	1.92 m		H-31
		1.83 m		H-19, H-30
7	122.2 CH	5.59 m	C: 9, 14	H-15, H-32
8	149.7 C			
9	48.1 CH	2.28 brd (12.8)		H-18, H-19
10	35.6 C			
11	22.8 CH_2_	1.63 m		
		1.38 m		H-32
12	34.8 CH_2_	1.96 m		H-17, H-21
		1.74 m		H-18
13	45.3 C			
14	52.9 C			
15	33.4 CH_2_	1.59 m		H-18
		1.50 m		H-7, H-32
16	22.2 CH_2_	1.93 m		H-23
		1.54 m		H-17, H-32
17	52.8 CH	2.39 t (8.8)	C: 14, 16, 18	H-21, H-32
18	24.8 CH_3_	1.28 s	C: 12, 14, 17	H-9, H-12, H-16, H-21
19	24.5 CH_3_	0.93 s	C: 1, 5, 9, 10	H-2, H-6, H-9
20	80.5 C			
21	24.5 CH_3_	1.62 s	C: 17, 20, 22	H-12, H-17, H-18
22	205.3 C			
23	119.9 C	7.41 d (15.5)	C: 22, 24, 25	H-17, H-21, H-26, H-27, H-16
24	156.5 C	7.40 d (15.5)	C: 22, 23, 25	
25	70.5 C			
26	29.3 CH_3_	1.47 s	C: 24, 25, 27	
27	29.2 CH_3_	1.48 s	C: 24, 25, 26	
30	17.4 CH_3_	1.02 s	C: 3, 4, 5, 31	H-2, H-6
31	28.7 CH_3_	1.17 s	C: 3, 4, 5, 30	H-3, H-5, H-6
32	30.7 CH_3_	1.03 s	C: 8, 13, 14, 15	H-7, H-11, H-12, H-15, H-17

^a^ Recorded at 176.04 MHz.

**Table 5:** NMR spectrosopic data (700 MHz, C_5_D_5_N/D_2_O (4/1 *v*/*v*)) of Aglycone Moiety of fallaxoside D_7_ (**4**).

Position	δ_C_ Mult. ^a^	δ_H_ Mult. (*J* in Hz)	HMBC	ROESY
1	36.4 CH_2_	1.60 m		H-11, H-19
		1.24 m		H-3, H-5, H-11
2	26.7 CH_2_	2.00 m		
		1.77 m		H-19, H-30
3	88.5 CH	3.10 dd (4.1; 11.8)		H-1, H-5, H-31, H-1 Xyl1
4	39.5 C			
5	49.3 CH	0.86 brd (13.7)		H-1, H-3, H-7, H-31
6	31.8 CH_2_	2.15 m		H-31
		1.79 m		
7	71.6 CH	3.83 m		H-5, H-32
8	49.8 CH	2.38 m		H-15, H-18, H-19
9	147.6 C			
10	38.9 C			
11	116.0 CH	5.27 m	C: 10, 13	H-1
12	35.8 CH_2_	2.34 m		H-32
		1.94 m		H-18
13	46.8 C			
14	47.3 C			
15	36.6 CH_2_	2.05 m		H-32
		1.94 m		
16	22.5 CH_2_	2.31 m		H-18
		1.62 m		H-32
17	59.3 CH	2.92 t (9.0)	C: 13, 18	H-12, H-32
18	16.4 CH_3_	0.59 s	C: 12, 13, 17	H-8, H-12, H-19
19	21.8 CH_3_	1.00 s	C: 5, 9, 10	H-1, H-2, H-8, H-18
20	212.5 C			
21	31.2 CH_3_	2.18 s	C: 17, 20	
30	16.6 CH_3_	0.94 s	C: 3, 4, 5, 31	H-2, H-31
31	27.9 CH_3_	1.14 s	C: 3, 4, 5, 30	H-3, H-5, H-6, H-30
32	18.3 CH_3_	0.99 s	C: 8, 13, 15	H-7, H-15, H-16, H-17

^a^ Recorded at 176.04 MHz
